# Fungal Polysaccharides as Modulators of Molecular Pathways in Liver Health

**DOI:** 10.3390/molecules30224384

**Published:** 2025-11-13

**Authors:** Rafał Szelenberger, Magdalena Więckowska

**Affiliations:** Biohazard Prevention Centre, Faculty of Biology and Environmental Protection, University of Lodz, Pomorska 141/143, 90-236 Lodz, Poland; magdalena.wieckowska@biol.uni.lodz.pl

**Keywords:** polysaccharide, β-glucan, mushroom, liver

## Abstract

Fungal polysaccharides represent a structurally diverse group of bioactive compounds with increasing recognition for their hepatoprotective potential. This review synthesizes current evidence on their roles in the prevention and treatment of liver diseases, including alcohol-related liver disease (ALD), metabolic dysfunction-associated fatty liver disease (MAFLD), or toxin-induced injury. The analyzed studies demonstrate that polysaccharides isolated from species such as *Lentinula edodes*, *Grifola frondosa*, *Ganoderma lucidum*, *Coriolus versicolor*, and *Cordyceps militaris* exert beneficial effects by reducing oxidative stress, attenuating inflammation, and improving metabolic homeostasis. Mechanistically, these effects are mediated through the regulation of multiple signaling pathways, including Nuclear Factor kappa-light-chain-enhancer of activated B cells (NF-κB), Nuclear factor erythroid 2–related factor 2 (Nrf2), and NOD-like receptor protein 3 (NLRP3) inflammasome, as well as modulation of gut microbiota. Fungal polysaccharides were also shown to improve hepatic function by lowering serum biomarkers of liver injury and ameliorating histopathological damage. Presented evidence indicates that fungal polysaccharides possess considerable potential as multifunctional hepatoprotective agents, highlighting the need for further mechanistic insight and clinical validation.

## 1. Introduction

The liver is an organ that performs a vast array of functions, including blood filtration and detoxification, nutrient processing, bile production, storage of vitamins and minerals, protein synthesis, regulation of blood glucose, metabolism of drugs and toxins, and immune function [1].

Various cell types contribute to the development of this multifaceted organ. Hepatocytes represent the main cell type (parenchymal cells) responsible for the liver’s essential functions, consisting of more than 80% of the total liver cell population. Other cell types (liver sinusoidal endothelial cells (LSECs), hepatic stellate cells (HSCs), Kupffer cells (KCs), cholangiocytes, and various immune cells), known as non-parenchymal cells, play a supporting role in maintaining liver homeostasis [2]. LSECs form the permeable, inner layer of the sinusoids, which are specialized capillaries that play a key role in the exchange of substances between the blood and liver cells. Moreover, LSECs (1) are central regulators of hepatic blood flow and pressure through the production of nitric oxide and other vasoactive molecules, maintaining sinusoidal tone and HSCs quiescence, (2) possess the highest endocytic activity among endothelial cells in the human body, enabling efficient clearance of blood-borne waste, viruses, and immune ligands via specialized receptors, (3) promote immune tolerance [3,4,5]. HSCs are located in the space of Disse and are responsible for (1) the storage of vitamin A, (2) modulation of immune response via secretion of anti-inflammatory molecules (i.e., chemokines and cytokines), (3) liver growth regulation mediated by the production of extracellular matrix components [3,6,7,8]. KCs are macrophages located within the sinusoids in the liver [6], which are associated with (1) bacteria engulfing via phagocytosis [9], (2) absorption and degradation of the immune complexes [10], (3) maintaining interaction with neutrophils and transferring captured pathogens to facilitate their disintegration [11], (4) production of vasodilatory carbon monoxide as a result of haemoglobin degradation [12], (5) recognition of danger signals like pathogen-associated molecular patterns (PAMPs) and/or damage-associated molecular patterns (DAMPs) released from injured hepatocytes and induction of the inflammasome and secretion of proinflammatory cytokines [3]. Cholangiocytes are epithelial cells located in the common bile duct and the intrahepatic bile ducts. Their main functions are bile modification and transport [13], and immunomodulation of the biliary system and liver by identifying microbial components and releasing the inflammatory modulators [14].

In 2023, it was estimated that liver diseases were responsible for approximately 2 million deaths annually, accounting for around 4% of all global mortality. Notably, 2 out of 3 liver deaths occur among men. Liver diseases represent a major threat to human health, as well as a significant economic burden due to the high cost of treatment and long-term care. The spectrum of liver disorders includes alcohol-associated liver disease (ALD), metabolic dysfunction-associated fatty liver disease (MAFLD), viral hepatitis, primary sclerosing cholangitis, primary biliary cholangitis, autoimmune hepatitis, acute liver failure, cirrhosis, and liver cancers [15].

In recent years, there has been a growing interest in the use of natural compounds as complementary agents in the prevention and treatment of various disease entities. This trend stems not only from the need to improve therapeutic efficacy and reduce drug-related side effects, but also from the complex role that natural bioactive compounds may play. Among these natural substances, polysaccharides draw attention due to their wide range of biological roles, including anti-inflammatory, antibacterial, antitumor, anticoagulant, antioxidant, antihypertensive, antihyperlipidemic, immunomodulatory, and hepatoprotective effects [16,17,18]. Given the global burden of MAFLD and ALD, and the limitations of conventional pharmacotherapy, the search for novel, multi-target therapeutic agents has intensified. Natural polysaccharides, with their potent anti-inflammatory properties and low systemic toxicity, represent a highly promising class of candidates [19].

One of the richest and most diverse sources of biologically active polysaccharides is fungi, especially edible and/or medicinal mushrooms, which produce a variety of structurally complex polysaccharides such as β-glucans, heteroglycans, and proteoglycans [20]. Fungal bioactive polysaccharides have a long history of use in traditional medicine and diet, particularly in Asia and parts of Eastern Europe. These biopolymers are valued for their immunomodulatory and antitumor properties, with some already developed into marketed therapeutics. Their medicinal benefits range from general health support and modulation of immune function to specific effects like anticancer, particularly among species of *Basidiomycetes*, and to a lesser extent, *Ascomycetes*, which have been traditionally used to treat gastrointestinal disorders and various cancers [21].

The ability of polysaccharides to modulate the gut microbiota may substantially contribute to their therapeutic effects, as this mechanism has been associated with improvements in various health conditions [22,23]. The gut-liver axis plays a central role in the development of liver diseases. Alterations in gut microbiota contribute to conditions such as MAFLD, hepatic encephalopathy, ALD, and hepatocarcinogenesis, mainly through increased intestinal permeability, systemic inflammation, and metabolic disturbances. Fungal polysaccharides, commonly present in the diet, exert protective effects on the liver. Their activity is largely attributed to prebiotic properties, including modulation of the gut microbiota composition, support of intestinal barrier integrity, regulation of microbiota-derived metabolites, reduction in inflammation and oxidative stress, and enhancement of the proper structure of the liver by attenuating the harsh effects of liver injury [24,25,26].

Therefore, this review aims to systematically analyze and summarize experimental evidence on the hepatoprotective effects of fungal polysaccharides, with a particular focus on the molecular pathways underlying their antioxidant and anti-inflammatory actions (NF-κB, Nrf2, and NLRP3). By organizing current data by mechanistic targets, this review provides an integrated framework linking fungal polysaccharides with their biological activity and liver health.

## 2. Methodology

The review was conducted following a structured literature search aimed to collect, summarize, and critically evaluate the available evidence on the hepatoprotective and molecular mechanisms of fungal polysaccharides, with particular emphasis on their involvement in NF-κB, Nrf2, and NLRP3 signaling pathways. A comprehensive literature search was limited to articles published in peer-reviewed journals and reputable scientific sources indexed in PubMed, Google Scholar, Scopus, Science Direct, and Web of Science databases to identify relevant studies published up to September 2025. The following keywords and their combinations were used: “fungal polysaccharides”, “mushroom polysaccharides”, “β-glucans”, “proteoglycans”, “homoglycans”, “protein-bound polysaccharides”, “heteroglycans”, “hepatocyte”, “liver injury”, “hepatoprotection”, “oxidative stress”, “Nrf2”, “NF-κB”, “NLRP3 inflammasome”, “gut-liver axis”, “hepatotoxicity”, “inflammation”, “pathways”. Studies were included if they investigated fungal-derived polysaccharides or their fractions, reported outcomes related to hepatoprotective, antioxidant, or anti-inflammatory effects, and provided information on molecular mechanisms or signaling pathways relevant to liver function or injury models. All records retrieved from databases were first screened for the title and abstract to remove duplicates and irrelevant papers, and later for the full text assessment for eligibility. After removing duplicates and irrelevant records, 23 studies met the inclusion criteria and were analyzed in detail. Given the diversity of study design and endpoints, a qualitative synthesis was performed instead of a meta-analysis. Extracted data were grouped thematically according to the predominant molecular pathways involved in hepatoprotection: (1) TLR4-NF-κB-mediated anti-inflammatory signaling, (2) Nrf2-mediated antioxidant response, and (3) NLRP3 inflammasome modulation. The final narrative summary was supported by schematic figures illustrating the major molecular mechanisms.

## 3. General Overview of Fungal Polysaccharides

Polysaccharides are naturally occurring carbohydrate biopolymers found in plants, animals, fungi, bacteria, and algae. Along with the other crucial biomacromolecules, proteins and nucleotides, polysaccharides are strongly associated with many biological processes [27]. They are composed of more than 10 monosaccharide units covalently linked with glycosidic bonds. Based on the monosaccharide unit composition, they can be divided into homopolysaccharide groups (with identical monosaccharide units) or heteropolysaccharides (with different monosaccharide units). Their physicochemical properties and biological functions are strongly determined by the monomer composition, degree of polymerization, branching patterns, and the type and position of glycosidic linkages [28].

An overview of selected fungal polysaccharides, along with their main properties, is provided in Table 1.

### 3.1. Homoglycans

Homoglycans are polysaccharides composed of repeating units of a single type of monosaccharide. In fungi, the most common homoglycans are β-glucans. β-glucans polymers are homoglycans composed of the β-D-glucose monomers, and are mainly found in the cell walls of plants, algae, bacteria, and fungi. They are a part of the endopolysaccharides (EnPs), which are also known as intracellular polysaccharides (IPS) [26,55]. β-glucans have a characteristic β(1→3)-linked D-glucopyranosyl backbone, although the length and branching pattern vary depending on the biological source. In fungi, β-glucans are typically branched and are distinguished by the presence of β(1→6)-glycosidic linkages that form side chains extending from the β(1→3) core. The conformation and length of the core and side chains are also associated with the fungal source and may be linked with the biological properties [59]. β-glucans, like other polymers, may change their conformation and adopt a shape similar to a sphere, rod, or worm; they can form a random coil and single-, double-, or triple-helix, and the last one appears to play a relevant role [60].

Lentinan, one of the most well-known β-glucans, is isolated from *Lentinula edodes*, commonly known as the shiitake mushroom. This species is naturally distributed across various regions, including Asia, Australia, North and South America, thriving particularly in warm and humid climates. It is one of the most widely consumed edible mushrooms, especially in Asian cuisine, which has contributed to its worldwide cultivation [61,62]. Numerous studies have established that *L. edodes* contains a diverse array of bioactive constituents with proven health benefits, including fiber, exogenous amino acids (i.e., histidine, methionine, phenylalanine, tryptophan, arginine), vitamins (i.e., thiamine, riboflavin, vitamin C, D, and E), minerals (i.e., Ca, K, Mg, Zn), and carbohydrates (including mono-, di- and polysaccharides) that constitute nearly 80% of the mushroom mass [63,64,65,66]. Lentinan belongs to the β-glucan family, and its structure consists of a β(1→3) backbone of D-glucose units with two β(1→6) D-glucosyl residues attached for every five D-glucose residues from the main chain [47]. In studies conducted by Zhang et al., it was demonstrated that lentinan can adopt different conformations depending on the used solution, i.e., in dimethyl sulfoxide, lentinan assumes a single random coil structure, whereas in sodium chloride, it adopts a triple-helical conformation [67,68,69]. Lentinan is mainly known for its antitumor activity, which was shown in 1969 in the Chihara et al. study [70]. Furthermore, in 1985, lentinan received approval in Japan for use as an adjuvant in the treatment of gastric cancer. Since then, it has been authorized for the management of various other malignancies such as hepatitis [71]. In the meta-analysis performed by Zhang et al., the response rates (RR) of the chemotherapy or chemotherapy with lentinan were summarized for lymphoma, lung, gastric, digestive system, gynecological, and breast cancers, and in patients with multiple cancers from 123 independent studies. Besides the fact that studies vary due to the different protocols and standards for defining RR, the addition of lentinan to the chemotherapy increased the efficacy and RR in patients who received only chemotherapy. For instance, in lung cancer, the increment ranged between 29 and 88.7%, for gastric cancer, 11.1–87.5%, and for hepatic cancer, 50.7–76% in comparison to 15.0–83.3%, 11.1–87.5, and 31.8–52% in only the chemotherapy group, respectively. Furthermore, Karnofsky performance status, which is an indicator used for the assessment of the patient’s functionality in oncology, showed a significant enhancement after lentinan administration [71]. The beneficial effect of lentinan was also confirmed in other in vitro and/or in vivo cancer model studies, including liver cancer [72,73,74], bladder cancer [75], and oral squamous cell carcinoma [76], and in other animal or experimental model diseases [77,78,79,80].

Pleuran is another β-glucan extracted from the fruiting bodies of *Pleurotus ostreatus*, one of the most extensively studied oyster mushrooms, which comprises over 200 species. The widespread culinary and nutritional relevance of *P*. *ostreatus* has driven its global cultivation, and it is frequently employed as a meat or fish substitute. Oyster mushrooms are characterized by a high content of protein, dietary fiber, and essential amino acids, particularly lysine and leucine, while exhibiting a low level of sodium and total fat. Notably, they also provide a valuable source of polyunsaturated fatty acids (PUFAs), including linolenic and linoleic acids, and are rich in biologically active polysaccharides with known health-promoting properties [81]. The structure of pleuran is characterized by a backbone of β-(1→3)-linked D-glucopyranosyl units. Every fourth glucose residue in the chain is substituted at the O-6 position with a single β-(1→6)- and β-(1→4)-linked internal residues [54]. Pleuran is also commercially available for supplementation in the form of syrup, named Imunoglukan P4H^®^ (Pleuran s.r.o., Bratislava, Slovakia) [82].

Its biological functions were tested and confirmed in studies examining various disease entities. Jesenak et al. performed a double-blind, placebo-controlled, randomised, multicentre study that evaluated the role of pleuran in the prevention of recurrent respiratory tract infections. Results showed that 36% of children who were administered Imunoglukan P4H^®^ did not suffer from infections of the respiratory tract in comparison to 21% of children who received a placebo (*p* < 0.05) [82]. Bergendiova et al. performed a double blind, placebo-controlled study to assess whether administration of Imunoglukan P4H^®^ would have a beneficial effect on upper respiratory tract infections in athletes over a training period. The obtained results showed that pleuran significantly decreased the occurrence of respiratory infections and elevated the number of natural killer cells in the bloodstream [83]. Urbancikova et al. performed a randomised, multicentre, double blind, and placebo-controlled study on 90 patients aged over 6 years with diagnosed herpes simplex facialis and/or labialis to investigate the role of Imunoglukan P4H^®^ on the duration of herpes symptoms. Results showed that administration of Imunoglukan P4H^®^ significantly reduced the number of days with herpes symptoms (*p* = 0.046). Although the 120-day supplementation with Immunoglukan P4H^®^ did not affect the incidence of infections, subjects receiving the supplement experienced a statistically significant reduction in the duration of rhinitis and cough (*p* = 0.05 and *p* = 0.024, respectively) [84]. In the study conducted by Bobek et al., the addition of 2% of dried fruiting bodies of *P. ostreatus* into the diet of male Syrian hamsters with chronic alcohol intake showed a significant reduction in the levels of cholesterol and triacylglycerols in serum and liver [85]. Moreover, water extract from *P. ostreatus* fruiting bodies helped in liver restoration in malnourished mice [86].

Another homoglycan is chitin. This polysaccharide is resistant to enzymatic digestion in the stomach, yet it undergoes microbial fermentation in the colon, producing beneficial metabolites [87]. Chitin deacetylases produced by fungi convert chitin into chitosan. This modification increases the polymer’s elasticity while reducing its immunogenicity and toxicity [88]. Chitosan is more soluble in the alkaline environment of the small intestine and is more susceptible to enzymatic action [87]. This compound is one of the most widely investigated polymeric carriers, valued for its non-toxicity, biocompatibility, low immunogenicity, biodegradability, and anti-microbial activity. Hence, this function is applied in the pharmaceutical fields [89]. Both biopolymers are only partially absorbed into the bloodstream, with smaller particles showing improved bioavailability. They exhibit multiple health-promoting effects, including anti-inflammatory and immunomodulatory properties, and cholesterol-lowering potential. By binding dietary fats, they can reduce lipid absorption, and their antimicrobial activity supports gut health by controlling pathogenic microbes and promoting beneficial microbiota [87]. One of the notable applications of chitosan is its use in the form of chitosan-based nanoparticles (ChNPs), which have emerged as promising nanomaterials with significant potential in drug delivery. These nanoparticles can encapsulate therapeutic agents or bioactive compounds, enabling targeted delivery and controlled release. In addition to their delivery capabilities, ChNPs exhibit notable anticancer properties, including demonstrated cytotoxicity against liver cancer cells [90]. Chitosan-based nanostructures can enhance the pharmacokinetics of both natural and synthetic drugs, improving the anti-hepatocellular carcinoma (HCC) effect. These nanoparticles are applicable in phototherapy, and the incorporation of targeting ligands such as arginylglycylaspartic acid (RGD) can increase drug accumulation in HCC cells. In addition, the chitosan-based nanocarriers, including reactive oxygen species (ROS)- and pH-sensitive nanoparticles, enable controlled release at the tumor site, maximizing HCC suppression [91].

### 3.2. Protein-Bound Polysaccharides and Proteoglycans

Proteoglycans are complex macromolecules composed of a core protein covalently linked to one or more glycosaminoglycan chains. They are a specialized class of glycoproteins distinguished by their high carbohydrate content and structural diversity. Proteoglycans are major components of the cell surfaces and extracellular matrix and play critical roles in cell signaling, tissue development, structural integrity, and even cancer initiation and/or progression [92]. In case of protein-bound polysaccharides, these compounds consist mainly of polysaccharides rather than proteins, do not contain classical glycosaminoglycans, and the protein fragments may be linked non-covalently [93,94].

Polysaccharopeptide (PSP) is a protein-bound polysaccharide extracted from *Trametes versicolor* (syn. *Coriolus versicolor*), a mushroom long valued in traditional Chinese medicine [57]. This fungus is widely distributed across temperate regions of Asia, North America, and Europe, including the United Kingdom [95]. PSP has an approximate molecular weight of 100 kDa. Its peptide fraction is rich in aspartic acid and glutamic acid, while the polysaccharide portion is composed of glucose with predominant α-1,4 and β-1,3 glucosidic linkages, and contains arabinose and rhamnose as distinguishing components [57]. The immunomodulatory and antitumor properties of PSP were discussed in the Dou et al. study with much effort, thus highlighting that PSP treatment improves quality of life and augments the effectiveness of chemotherapy, radiotherapy, and overall pharmacology in patients with cancers, liver diseases, hyperlipidemia, chronic bronchitis, and other long-term conditions [93].

Similarly to PSP, Polysaccharide-K (PSK) is a protein-bound polysaccharide also isolated from *Trametes versicolor*. PSK has an approximate molecular weight of 94 kDa and consists of about 25% peptides and 75% carbohydrates. The peptide fraction is rich in glutamic acid, aspartic acid, leucine, and valine, with smaller contributors from threonine, serine, and glycine. The carbohydrate fraction is dominated by glucose, accompanied by minor amounts of mannose, fucose, xylose, and galactose. Structurally, PSK contains a β-glucan backbone with (1→4) linkages, branched at the 3- and 6-positions, and incorporates (1→3) and (1→6) glucopyranosidic bonds. Biochemical analyses have shown the presence of two high-molecular-weight glycosylated forms, which can be processed into 12 kDa fragments after neuraminidase digestion. PSK is one of the best-known mushroom-derived polysaccharides with well-documented anticancer properties [56]. Yunoki et al. reported that PSK exerts hepatoprotective and antitumor effects. Oral administration of PSK enhanced hepatic natural killer (NK) cell activity, particularly within low-density lymphocyte fractions, thereby contributing to increased cytotoxicity against tumor cells. These findings indicate that PSK may support the prevention of liver metastasis through the activation of organ-associated immune response [96]. It has demonstrated significant activity in both in vitro and clinical trials, making it a prominent example of how natural products can contribute to cancer therapy [56].

Yang et al. conducted a study to evaluate the effects of a proteoglycan isolated from the mycelia of *Ganoderma lucidum* (GLPG) on C-C Motif Chemokine Ligand 4 (CCL_4_)-induced liver injury. The results demonstrated that GLPG supplementation reduced the extent of L-02 cell damage, lowered plasma levels of alanine aminotransferase (ALT), aspartate aminotransferase (AST), and tumor necrosis factor α (TNFα), and enhanced superoxide dismutase (SOD) activity, indicating a significant role in the reduction in oxidative stress [41].

### 3.3. Heteroglycans

Heteroglycans, also known as heteropolysaccharides, are composed of two or more different types of monosaccharides. Unlike homoglycans, which contain only a single sugar unit, heteroglycans have a diverse and often branched structure, allowing them to interact with multiple biological targets [28].

Polysaccharide fraction isolated from *Dictyophora indusiata* (DIP) has been classified as a heteroglycan composed of glucose (53.25%), galactose (24.18%), mannose (19.19%) and xylose (3.37%) [33]. The protective role of DIP against sodium arsenite-induced injury in human hepatic L-02 cells was investigated. The finding demonstrated that DIP pretreatment effectively protected hepatocytes from arsenic-induced apoptosis nu upregulating the anti-apoptotic protein B-cell lymphoma 2 (Bcl-2) and downregulating the pro-apoptotic Bcl-2-associated X protein (Bax). Proteomic analysis showed that DIP restored arsenic-disrupted expression of metabolism- and apoptosis-related proteins, indicating its hepatoprotective effect [97]. Furthermore, DIP can scavenge intracellular ROS, thereby reducing oxidative stress and suppressing PTEN-induced kinase 1 (PINK1)/Parkin pathway activation, which prevents mitochondrial autophagy and mitigates arsenic-induced hepatotoxicity [98]. DIP was also investigated in Hu et al.’s study on the HCC in vitro model. Results showed that DIP inhibited proliferation in a time- and dose-dependent manner, induced G2/M phase arrest, and promoted apoptosis. Furthermore, DIP upregulated pro-apoptotic markers, including BAX and caspase-3, suggesting its anticancer activity through cell cycle regulation and apoptosis induction [99]. In a Sprague-Dawley rat model of arsenic-induced liver injury, DIP treatment reversed alterations in metabolic, mitochondrial, oxidative stress, and apoptosis-related proteins, mitigating hepatic damage [100]. Polysaccharide extract from *Fomitopsis pinicola* was also considered as a heteroglycan consisting of myo-inositol, fucose, galactose, glucose, mannose, and fructose [37]. *Fomitopsis pinicola* mycelial polysaccharides (FPMPS) restored normal hepatic and cecal morphology and reversed alcohol-induced alteration in serum lipid levels in a murine model of ALD. Furthermore, FPMPS was found to modulate retinol metabolism, bile acid secretion, and the phosphoinositide 3-kinase (PI3K)-protein kinase B (Akt) signaling pathway. The authors concluded that FPMPS supplementation exhibits significant hepatoprotective effects, highlighting its potential as a therapeutic agent for ALD [36].

## 4. Effect of the Fungal Polysaccharide for Hepatoprotection, Antioxidant Activity, and Anti-Inflammation Processes Through Regulation of TLR4/NF-κB Pathway

Mushrooms are a source of bioactive compounds that collectively exert a consistent set of biological activities relevant to liver health: antioxidant activity, modulation of inflammatory signaling, immune regulation, regulation of lipid/glucose metabolism, and modulation of gut microbiota with downstream effects on the gut-liver axis. These combined activities underlie the hepatoprotective effects observed across models of drug-induced liver injury, alcoholic and non-alcoholic fatty liver disease, fibrosis, and certain carcinogenesis models [16,26,101].

Gut-derived endotoxins, especially lipopolysaccharides (LPS) derived from Gram-negative bacteria, contribute to the development of MAFLD. Dysbiosis and intestinal barrier dysfunction facilitate bacterial translocation and allow LPS to enter the portal circulation, activating hepatic pattern recognition receptors (PRRs), including Toll-like receptor 4 (TLR4) and inflammasomes [102,103]. This process triggers Nuclear Factor kappa-light-chain-enhancer of activated B cells (NF-κB) activation and overproduction of pro-inflammatory cytokines, promoting chronic hepatic inflammation and disease progression [19]. Thus, therapeutic interventions aimed at restoring gut homeostasis, strengthening intestinal barrier integrity, and attenuating LPS-driven signaling represent promising strategies in MAFLD management [104,105]. In the quiescent state, NF-κB is retained in the cytosol through its binding to the inhibitory protein—IκB, which prevents its nuclear translocation. Upon activation, κB is rapidly phosphorylated and degraded. This releases the active Rel-proteins, which then translocate to the nucleus, bind to consensus DNA elements, and initiate the transcription of a cascade of inflammatory genes. Consequently, inhibition of NF-κB activation is the primary mechanism by which many polysaccharides exert their anti-inflammatory effects [19].

Accumulating evidence suggests that mushroom-derived polysaccharides exert hepatoprotective effects through precisely these mechanisms. In high-fat diet-fed (HFD) mice, *Hirsutella sinensis* polysaccharides (HSP) significantly reduced body weight, insulin resistance, and visceral fat, while lowering serum triglycerides and modulating lipid metabolism genes in hepatic and adipose tissues. Importantly, supplementation decreased fasting glucose, insulin, pro-inflammatory cytokines (IL-1β, TNFα), and circulating endotoxin levels, while restoring gut microbiota composition. Histological analysis revealed reduced steatosis and inflammation, indicating protection against both MAFLD and Non-Alcoholic Steatohepatitis (NASH) [43].

Similarly, polysaccharides from *Grifola frondosa* (GFP) demonstrated robust hepatoprotective activity in HFD-induced MAFLD rats. GFP treatment improved liver enzymes and lipid profile, while enhancing antioxidant defense (SOD, glutathione peroxidase (GPH-Px)). Mechanistically, GFP downregulated cytochrome P450 (*Cyp4a1*), implicated in lipid ω-oxidation and hepatocellular injury, while upregulating *Cyp7a1*, thereby promoting cholesterol clearance via bile acid synthesis. Moreover, GFP suppressed pro-inflammatory and lipogenic genes (acetyl-CoA carboxylase (*Acc*), *Tnfα*, Suppressor of cytokine signaling 2 (*Socs2*)) and favorably reshaped gut microbiota, collectively mitigating steatosis and hepatic injury [106]. Similar results for GFP were obtained by Li et al. [107]. Furthermore, it has been demonstrated that in a rat model of type 2 diabetes, supplementation with GFP resulted in a statistically significant decrease in IL-1β, TNFα, Monocyte Chemoattractant Protein-1 (MCP-1), and TLR4, accompanied by an increase in IL-10 and AMP-activated protein kinase (AMPK) in the liver [108]. Currently, there is no conclusive evidence that GFP directly modulates the TLR4/NF-κB pathway. This effect has been demonstrated in the kidney in early diabetic nephropathy [109]; however, observations indicating a reduction of TLR4 in the liver upon GFP administration suggest that a similar mechanism may also be present in this organ.

Chen et al. investigated GLPG in a CCL_4_-induced liver fibrosis mouse model. GLPG supplementation significantly lowered ALT, AST, and lactate dehydrogenase (LDH), indicating protection against CCl_4_-induced hepatic injury. Histopathological examinations revealed decreased hepatocyte infiltration and overall improvement of liver tissue structure. Moreover, GLPG suppressed HSC activation and downregulated the expression of pro-inflammatory cytokines such as TNFα, IL-1β, and IL-6, in both plasma and liver tissues. Similar results were obtained in Chen et al. study [110]. Further investigations focused on the possible mechanism of action demonstrated that GLPG inhibited activation of the TLR4/Myeloid differentiation primary response 88 (MyD88)/NF-κB signaling pathway induced by CCL_4_, suggesting a potent anti-inflammatory and anti-fibrotic effect in hepatocytes [110]. Similar conclusions were drawn by Zhang et al., who investigated whether GLPG could attenuate APAP-induced liver injury. Administration of GLPG resulted in more than a two-fold reduction in serum AST and ALT levels, compared to APAP-treated group. Histopathological analyses showed that GLPG improved liver tissue architecture, reduced infiltration of pro-inflammatory cells and necrolysis, normalized hepatocyte morphology, and diminished apoptosis. Moreover, GLPGs alleviated APAP-induced oxidative stress by lowering hepatic MDA and ROS levels, while significantly increasing the activity of SOD and GSH-Px, and the level of GSH [111]. Comparable benefits were also reported for polysaccharides from the water extract of *Ganoderma lucidum* (WEGL), which improved lipid metabolism, reduced oxidative stress, and attenuated inflammatory signaling [58].

Lentinan further highlights the immunometabolic, antioxidant, and anti-inflammatory potential of fungal polysaccharides. In the study conducted by Yang et al., administration of lentinan in the arsenic-induced hepatotoxicity mice model, attenuated the expansion of hepatic sinusoids and reduced hepatocyte edema, as observed in histopathological analyses. Furthermore, it significantly decreased plasma levels of ALT and AST, thereby improving overall liver tissue condition. Moreover, lentinan supplementation markedly reduced hepatic MDA levels while enhancing GSH content [112]. In HFD-fed mice, lentinan reduced hepatic inflammation and glucose intolerance by suppressing NF-κB activation and Protein Tyrosine Phosphatase 1B (Ptp1b) expression, thereby restoring insulin signaling *via* the Akt–glycogen synthase kinase 3 beta (GSK3β) cascade. Lentinan also downregulated hepatic Lipopolysaccharide Binding Protein (*Lbp*) and *Tlr4*, decreased pro-inflammatory cytokines and chemokines (*Tnfα*, *Il-6*, *Il-1β*, and *Mcp-1*) and reduced macrophage infiltration in the liver. Transcriptomic analyses confirmed modulation of PI3K-Akt and cytokine–cytokine receptor pathways, linking metabolic and immune regulation [113].

In a HFD mouse model of MAFLD, *Coriolus versicolor* polysaccharide (CVP) at 400 mg/kg reduced serum and hepatic triglyceride and cholesterol levels showing a strong anti-lipidemic effect. It also improved gut microbiota composition [32]. In the study conducted by Wang et al., polysaccharide PSP-1b1, a fraction of PSP, was investigated for its ability to improve liver condition in a mouse model of ALD. Supplementation with PSP-1b1 significantly reduced plasma ALT and AST levels. Histopathological analysis revealed a decrease in fatty deposits and necrosis compared with the alcohol-treated group. To elucidate the potential mechanisms underlying PSP-1b1 activity, the authors analyzed the expression of genes related to oxidative stress, lipid peroxidation (LPO), and the TLR4 pathway in the liver. The results demonstrated that PSP-1b1 markedly downregulated the expression of *Tlr4*, *Myd88*, Cluster of Differentiation 14 (*Cd14*), *Il-1β*, *Tnfα*, *Cyp2e1*, Inducible Nitric Oxide Synthase (*iNos*), and Heme Oxygenase-1 (*Ho-1*), while upregulating *Sod*, catalase (*Cat*), and Peroxisome Proliferator-Activated Receptor α (*Pparα*). At the protein level, significant reductions were observed in p-AMPKα/AMPKα, Cyp2E1, MyD88, and TNFα expression. These findings suggest that PSP-1b1 supplementation promotes antioxidant processes to protect the liver from damage by reducing oxidative stress and LPO, in which Cyp2E1 plays a key role. Furthermore, PSP-1b1 exhibited immunomodulatory properties by markedly suppressing the expression of genes and proteins involved in the TLR4 signalin pathway [114]. Similar results were obtained for PSP in Ren et al.’s study, including its important role in endotoxin-triggered TLR4/NF-κB signaling pathway in the liver of ALD-induced mice [115].

In a CCl_4_-induced mouse model of liver injury, Zhang et al. showed that Auricularia auricula polysaccharides (AAP) markedly reduced hepatic fibrosis and inflammatory infiltration. The treatment also decreased serum AST and ALT levels, confirming their hepatoprotective potential. Moreover, transcriptomic analyses revealed that AAP-treated mice exhibited reduced mRNA expression of *Tnfα*, Interferon γ (*Ifnγ*), *Il-6*, Cyclooxygenase-2 (*Cox2*), *iNos*, and *Myd88* compared with mice treated with CCL_4_ alone. Decreased protein levels of TLR4, phosphorylated Inhibitor of kappa B alpha (p-IκBα), and p-NF-κB were observed, indicating that AAP can suppress inflammatory responses and inhibit the overactivation of TLR4/NF-κB signaling pathway [29].

Intracellular mycelium polysaccharides from *Pleurotus geesteranus* (IMPP) exhibited a pronounced hepatoprotective and antioxidant activity. Treatment with lowered hepatic injury markers (ALT, AST, and alkaline phosphatase (ALP))and oxidative enzymes (Cyp2E1 and myeloperoxidase (MPO)) activity, while enhancing detoxifying enzymes (alcohol dehydrogenase (ADH) and acetal dehydrogenase (ALDH)). It also suppressed inflammatory cytokines (TNFα, IL-1β and IL-6) and reduced lipid peroxidation indicators (malondialdehyde (MDA) and LPO). In parallel, IMPP markedly increased the activities of hepatic antioxidant enzymes, including SOD, glutathione peroxidase (GSH-Px), CAT, and Total Antioxidant Capacity (T-AOC) [46]. In Song et al.’s study, a supplementation of polysaccharide isolated from *Pleuroteus geestranus* named PFP-1 increased hepatic activities of ADH and ALDH while reducing Cyp2E1 activity compared with alcohol-only treated mice. PFP-1 treatment significantly decreased hepatic ROS and MDA levels and enhanced the activities of antioxidant enzymes, including SOD, GSH-Px, and CAT, in ALD mice. Moreover, PFP-1 was shown to induce the TLR4/NF-κB pathway [51].

A significant enhancement of hepatic antioxidant and anti-inflammatory properties was also shown for *Antrodia cinnamomea* polysaccharide (ACP). In a study using slow-growing broiler breeds stimulated with LPS, ACP administration significantly increased the activities of T-AOC, GSH-Px, and T-SOD, and decreased the level of MDA, TNFα, IL-1β, and IL-6. Moreover, ACP markedly downregulated activation of the hepatic TLR4/NF-κB signaling pathway [30]. Improvement of liver function, both in serological and histopathological assessments, has also been demonstrated for PCP in a rat model of ALD. The study showed that PCP supplementation positively modulated ferroptosis, lipid peroxidation, and oxidative stress by increasing hepatic levels of GSH and SOD, while reducing MDA and Fe^2+^ concentrations. Moreover, PCP treatment significantly decreased serum inflammatory factors, including IL-1β, IL-6, LPS, and TNFα, compared with untreated ALD rats. Gene expression analysis in the liver following PCP supplementation revealed a significant downregulation of *NF-κB*, *Myd88*, *Il-1β*, and *Il-6*. Moreover, the hepatic protein levels of MyD88, IL-1β, and IL-6 were also markedly reduced, indicating that PCP exerts its hepatoprotective effect by modulating NF-κB signaling pathway [116].

Enzymatic-extractable polysaccharides from *Cordyceps militaris* (EPCM) were investigated in a CCL_4_-induced liver injury mouse model. In the study conducted by Zhao et al., EPCM demonstrated strong antioxidant activity, including significant reducing power and scavenging capacity against hydroxyl radicals and 2,2-diphenyl-1-picrylhydrazyl (DPPH). Histopathological analysis revealed that EPCM improved liver tissue architecture. Furthermore, mice treated with EPCM exhibited enhanced antioxidant defense, as evidenced by increased activities of SOD, CAT, and GSH-Px, along with decreased MDA levels, compared with mice exposed to CCL_4_ alone. The effects of EPCM on the NF-κB signaling pathway were also evaluated. EPCM supplementation significantly reduced hepatic levels of TNFα, IL-6, and COX-2, while markedly increasing IκBα expression and reducing the ratio of phosphorylated IκBα and phosphorylated NF-κB, p65 subunit (p-NF-κB p65)/NF-κB p65. Immunohistochemical analysis further confirmed these findings, supporting the role of EPCM in attenuating inflammation and oxidative stress via modulation of the NF-κB pathway [35].

Teng et al. investigated the hepatoprotective potential of *Morchella esculenta* polysaccharide 2 (MEP2) in a murine model of ALD. Similarly to other mushroom-derived polysaccharides, MEP2 administration significantly reduced serum levels of AST, ALT, Gamma-Glutamyl Transferase (GGT), and hepatic Cyp2E1, while concomitantly increasing ADH and ALDH activities. Histopathological analyses further confirmed that MEP2 supplementation reversed pathological alterations, including inflammatory cell infiltration, reduced hepatocyte nuclear density, lipid vacuole accumulation, hepatocellular swelling, and disorganization of hepatic cords. Moreover, MEP2 supplementation significantly enhances the concentration of antioxidant enzymes, including GSH-Px, SOD, and CAT, while concurrently decreasing hepatic ROS and MDA levels. Importantly, MEP2 treatment inhibited NF-κB pathway activation and decreased the expression of its downstream proteins in the liver. Moreover, MEP2 markedly reduced the hepatic levels of interferon α (IFNα), IFNγ, TNFα, IL-6, and IL-16, while simultaneously enhancing the expression of IL-7, IL-10, and IL-22 [49]. Of note, IL-10 has been reported to downregulate TNFα and IFNγ gene expression in injured liver and to modulate tissue injury through its regulatory effects on T cells and macrophages [117]. In the case of IL-7, it has been demonstrated that hepatocytes are capable of producing substantial amounts of this cytokine in response to TLR activation, suggesting that MEP2 may induce NF-κB signaling through TLR-mediated pathways [118]. Moreover, hepatoprotective properties have also been demonstrated for IL-22, which enhances the innate immunity of tissue cells, protects them from injury, and promotes regeneration. Importantly, IL-22 is a well-documented antioxidant factor in hepatocytes, acting through the induction of genes involved in oxidative stress defense [119].

At the level of the gut–liver axis, mushroom polysaccharides consistently improve intestinal barrier function and reduce endotoxemia. GFP supplementation has been shown to correct HFD-induced dysbiosis by decreasing the *Firmicutes*-to-*Bacteroidetes* ratio, as well as reducing abundances of *Acetatifactor*, *Alistipes*, *Flavonifractor*, *Paraprevotella*, and *Oscillibacter*. In parallel, a significant increase was observed in the population of *Allobaculum*, *Bacteroides*, *Bifidobacterium*, *Blautia*, and *Coprococcus* [38]. Similarly, treatment with WEGL, CVP, AAP, and DIP also decreased the *Firmicutes*-to-*Bacteroidetes* ratio [29,32,58,120]. Interestingly, the upregulated ratio was observed for ACP [30]. What is more, WEGL also reduced the *Proteobacteria* phylum, frequently expanded in MALD and associated with elevated endotoxin load [58]. Further, lentinan supplementation restored microbial richness and composition in comparison to the HFD-induced MAFLD mouse model by reducing *Proteobacteria* and *Epsilonbacteraeota* and augmenting *Firmicutes* and *Actinobacteria*, including *Bifidobacterium* [113]. Beyond reshaping gut microbial composition, mushroom polysaccharides exert a profound effect on intestinal barrier integrity and systemic endotoxemia. HSP, WEGL, and lentinan markedly reduced serum LPS concentrations [43,58,113]. Mechanistically, WEGL supplementation restored tight junction proteins zonula occludens-1 (ZO-1) and occludin, thus reinforcing intestinal barrier integrity. Additionally, WEGL decreased hepatic TLR4 protein levels, suppressed c-Jun N-terminal kinase (JNK) phosphorylation, and stabilized IκB-α, thereby preventing NF-κB nuclear translocation and dampening hepatic inflammation [58]. HSP likewise enhanced intestinal ZO-1 expression, strengthening barrier function and further mitigating endotoxin-driven inflammation [43]. Lentinan supplementation not only reduced systemic LPS levels but also improved intestinal barrier integrity through upregulation of ZO-1 and occludin expression at both the transcriptional and translational levels [113]. Similar results were obtained for PSP-1b1 [114]. The aforementioned findings therefore suggest that mushroom-derived polysaccharides possess the ability to modulate the activity of the TLR4-NF-κB pathway and reduce the inflammation in the liver. Empirical evidence unequivocally indicates that fungal polysaccharides act through a multi-directional strategy to promote liver health. This strategy includes restoring a healthy composition of the gut microbiota, strengthening the integrity of the intestinal barrier, and significantly lowering circulating LPS levels. The key molecular mechanisms associated with the action involve the modulation of the TLR4/NF-kB pathway, leading to the inhibition of inflammation and attenuation of oxidative stress.

**Figure 1:**
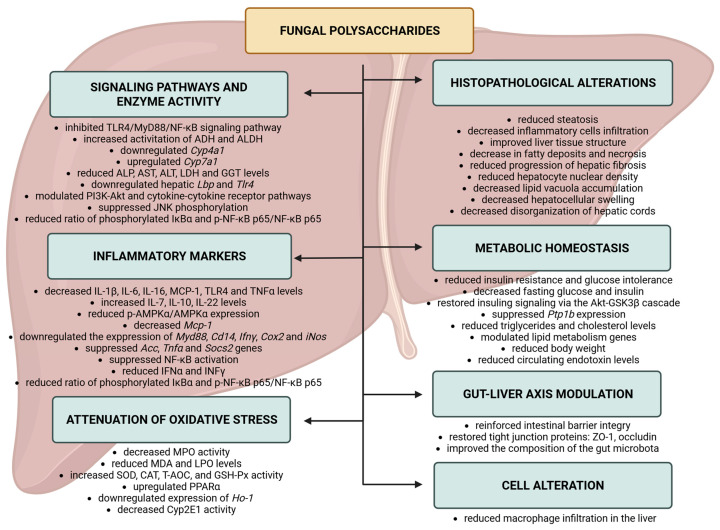
Effects of the fungal polysaccharide processes through regulation of TLR4/NF-κB pathway. Abbreviations: Acc: Acetyl-CoA Carboxylase; ADH: alcohol dehydrogenase; Akt: The protein kinase B; ALDH: acetal dehydrogenase; ALP: alkaline phosphatase; ALT: alanine aminotransferase; AMPK: AMP-activated protein kinase; AST: aspartate aminotransferase; CAT: catalase; Cd14: Cluster of Differentiation 14; Cox2: Cyclooxygenase-2; Cyp: cytochrome P450; GGT: Gamma-Glutamyl Transferase; GSH-Px: glutathione peroxidase; GSK3β: cascade glycogen synthase kinase 3 beta; HO-1: Heme Oxygenase-1; IFN: interferon; IL: interleukin; iNos: Inducible Nitric Oxide Synthase; IκBα: inhibitor of κBα; JNK: c-Jun N-terminal kinase; Lbp: Lipopolysaccharide Binding Protein; LDH: lactate dehydrogenase; LPO: lipid peroxidation; Mcp-1: monocyte chemoattractant protein-1; MDA: malondialdehyde; MPO: myeloperoxidase; MyD88: Myeloid differentiation primary response 88; NF-κB: Nuclear Factor kappa-light-chain-enhancer of activated B cells; PI3K: Phosphoinositide 3-Kinase; p-NF-κB p65: phosphorylated NF-κB, p65 subunit; PPARα: Peroxisome Proliferator-Activated Receptor Alpha; Ptp1b: Protein Tyrosine Phosphatase 1B; Socs2: Suppressor of Cytokine Signaling 2; SOD: superoxide dismutase; T-AOC: Total Antioxidant Capacity; TLR4: Toll-like receptor 4; Tnfα: Tumor Necrosis Factor alpha; ZO-1: zonula occludens-1. Created in BioRender. Bijak, M. (2025) https://BioRender.com/0iwpkmp (accessed on 27 October 2025).

Studies have consistently shown that these compounds improve liver function by reducing the concentration of liver enzymes (ALT, AST), increasing the activation of detoxification enzymes (ADH and ALDH), and mitigating pathological changes such as steatosis, inflammatory cell infiltration, and the progression of hepatic fibrosis (Figure 1). This complex and multifaceted mechanism of action suggests that fungal polysaccharides represent valuable adjunctive or alternative therapeutic candidates for the management of chronic liver diseases.

## 5. Effect of the Fungal Polysaccharide for Hepatoprotection, Antioxidant Activity, and Anti-Inflammation Processes Through Regulation of Nrf2 Pathway

Nuclear factor erythroid-2-related factor 2 (Nrf2) is a master transcription factor regulating cellular antioxidant defenses and cytoprotective responses in the liver [121]. Under basal conditions, Nrf2 is bound to its inhibitor Keap1 in the cytoplasm, which facilitates its ubiquitination and proteasomal degradation. Upon oxidative stress or exposure to bioactive compounds, such as fungal polysaccharides, Keap1 undergoes conformational changes, allowing Nrf2 to translocate into the nucleus. In the nucleus, Nrf2 binds to antioxidant response elements (AREs) in the promoters of target genes, inducing transcription of cytoprotective enzymes including HO-1, SOD, CAT, GSH-Px, and NAD(P)H quinone oxidoreductase 1 (NQO1) [122]. Activation of these downstream targets enhances hepatic antioxidant capacity, reduces reactive oxygen species (ROS), limits lipid peroxidation, and mitigates hepatocyte apoptosis during liver injury. Furthermore, Nrf2 activation preserves hepatocyte integrity, stabilizes mitochondrial function, and alleviates inflammation, in part through cross-talk with NF-κB and AMPK pathways [123].

In a study conducted by Zhao et al., the hepatoprotective potential of *Phellinus linteus* polysaccharides (Phps) and their ability to activate the AMPK/Nrf2 signaling pathway were evaluated in a murine model of acetaminophen (APAP)-induced acute liver injury. The administration of Phps significantly reduced plasma levels of AST and ALT, and markedly increased hepatic glutathione (GSH) content while decreasing MPO activity. Histopathological examination revealed a pronounced reduction in necrotic hepatocytes and improved tissue architecture. Mechanistically, APAP exposure was found to suppress the expression of proteins involved in the AMPK pathway, including phosphorylated ACC, AMPKβ, AKT, and AMPKα. Treatment with Phps effectively reversed these alterations, leading to a significant increase in phosphotylation of ACC, AMPKβ, AKT, and AMPKα. Additionally, Phps enhanced Nrf2 nuclear translocation and upregulated the expression of its downstream antioxidant targets, including NQO1, HO-1, Glutamate–Cysteine Ligase Modifier Subunit (GCLM), and Glutamate–Cysteine Ligase Catalytic Subuni (GCLC). Importantly, the critical role of Nrf2 in mediating these effects was confirmed using Nrf2^-/-^ knockout mice. In the absence of Nrf2, Phps supplementation failed to reduce liver injury markers (AST and ALT), restore antioxidant protein expression, or improve histopathological features. These findings demonstrate that the hepatoprotective effects of Phps are predominantly dependent on the activation of the AMPK/Nrf2 signaling pathway [121]. Regulation of AMPK has also been demonstrated for PSP and *Morchella esculenta* polysaccharide (MCP) [48,115]. The authors suggest that MCP primarily acts via the AMPK/sirtuin 1 (Sirt1) pathway; however, AMPK is also a well-known activator of the Nrf2 signaling pathway. Therefore, it is highly plausible that PSP and MCP could modulate the activity of both pathways through AMPK activation, although confirmation of this hypothesis would require more detailed mechanistic studies [48,124]. Similar results were obtained for GLPG, whose supplementation markedly upregulated Nrf2 and its downstream targets, including HO-1, GCLC, and NQO1, at both the protein and mRNA levels [111].

In a study conducted by Yang et al., the hepatoprotective effects of lentinan were evaluated in mice exposed to arsenic. Study showed that arsenic exposure alone increased hepatic Nrf2 and NQO1 levels; however, lentinan administration further amplified their expression by approximately 1.6- and 1.4-fold, respectively, indicating a potentiation of the Nrf2-mediated antioxidant response [112]. Evidence for lentinan-mediated activation of the Nrf2 pathway and its downstream targets has also been demonstrated in HK-2 [125] and BMECs [126] cell lines, and in the small intestinal tissue of HFD-induced mice [113].

Gene and protein expression analyses revealed a significant upregulation of Nrf2 and its downstream target HO-1 in the liver following PCP supplementation. Furthermore, the expression of genes and proteins involved in oxidative stress regulation, including Glutathione Peroxidase 4 (GPx4) and GSH, was also markedly enhanced, highlighting the hepatoprotective potential of PCP through activation of the Nrf2-mediated antioxidant response [116]. Similar results were also found for PFP-1 [46,51]. Western blot and immunofluorescence analyses confirmed that PFP-1-treated ALD mice exhibited elevated levels of Nrf2 and its downstream target HO-1 compared with untreated controls [51].

Polysaccharides isolated from *Cordyceps militaris* have also been shown to modulate the Nrf2 signaling pathway. In addition to the ability of EPCM to regulate the NF-κB pathway, as discussed above, Zhao et al. further evaluated the concentration of Nrf2 and HO-1. Their findings demonstrated that EPCM supplementation in mice with CCL_4_-induced liver injury significantly increased the expression of these proteins compared with untreated animals, suggesting that the antioxidant properties of EPCM may be mediated through mechanisms associated with the Nrf2 pathway [35].

The regulatory effect of MEP2 on the Nrf2 signaling pathways has also been suggested in a murine model of ALD. Both immunohistochemistry and Western blot analyses demonstrated that MEP2 treatment upregulated the expression of Nrf2 and its downstream targets, such as HO-1, NQO1, SOD-1, and CAT, in the livers of alcohol-induced mice [49].

**Figure 2:**
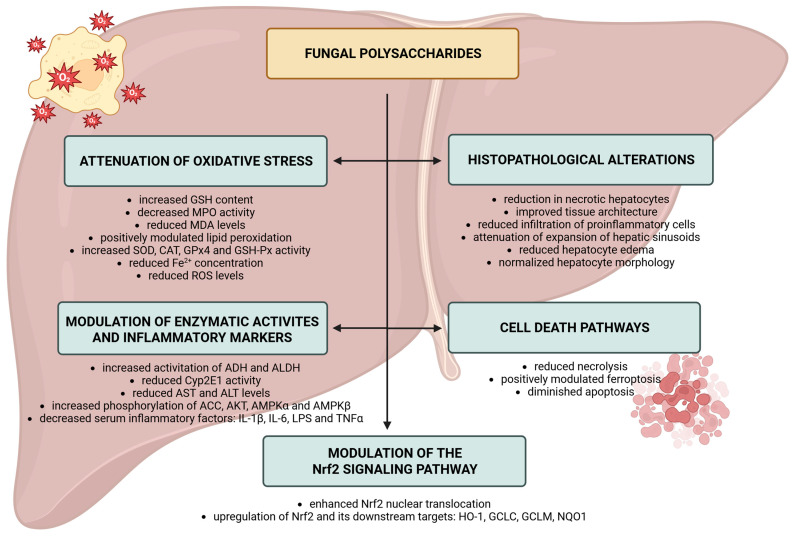
Effects of the fungal polysaccharides through regulation of Nrf2 pathway. Abbreviations: Cyp2E1: cytochrome P450 2E1; GCLC: Glutamate–Cysteine Ligase Catalytic Subunit; GCLM: Glutamate–Cysteine Ligase Modifier Subunit; GPx4: glutathione peroxidase 4; GSH: glutathione; LPS: lipopolysaccharide; NQO1: NAD(P)H quinone oxidoreductase 1; Nrf2: Nuclear factor erythroid 2–related factor 2; ROS: reactive oxygen species. Created in BioRender. Bijak, M. (2025) https://BioRender.com/5ki3q1l (accessed on 27 October 2025).

The aforementioned studies conclusively highlight the critical role of fungal polysaccharides in regulating the Nrf2 pathway, thereby demonstrating a significant influence on the modulation of Nrf2 and its downstream targets. Moreover, the polysaccharides mentioned above exhibit a substantial antioxidative effect by increasing the levels of GSH and the activity of key enzymes such as SOD, CAT, GPx4, concurrently reducing the levels of MDA and the activity of MPO. Furthermore, decrease the markers of oxidative stress, modulate the processes of cell death, and positively influence hepatocyte morphology by mitigating structural damage and supporting reparative processes, ultimately leading to the normalization of liver tissue architecture (Figure 2).

## 6. Effect of Fungal Polysaccharides on the NLRP3 Inflammasome

The NOD-like receptor protein 3 (NLRP3) inflammasome is a multiprotein complex that plays a pivotal role in liver immunity and inflammation. It is primarily expressed in Kupffer cells, hepatic stellate cells, and hepatocytes under stress conditions, and its activation is triggered by various DAMPs and PAMPs, including LPS and ROS. Upon activation, NLRP3 recruits the apoptosis-associated speck-like protein containing a caspase recruitment domain CARD (ASC) and procaspase-1, leading to the formation of active caspase-1, which subsequently processes pro-inflammatory cytokines, IL-1β, and IL-18 into their mature, bioactive forms. This cascade promotes inflammation and hepatocyte injury, and can exacerbate fibrosis in chronic liver diseases [127]. The involvement of polysaccharides in regulating NLRP3 inflammasome activity in various cell types and models has been demonstrated, e.g., Agaricus *blazei* Murill [128], *Hericium erinaceus* [129] and *Inonotus obliquus* [130]. However, the number of studies addressing the effects of mushroom-derived polysaccharides on inflammasome activity in the liver remains limited.

**Figure 3:**
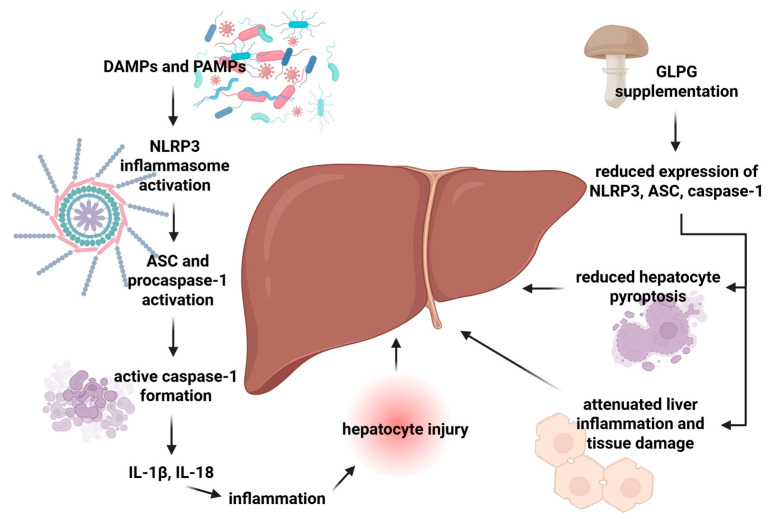
Effects of GLPG on the NLRP3 inflammasome. Abbreviations: ASC: apoptosis-associated speck-like protein containing a caspase recruitment domain CARD; DAMPs: damage-associated molecular patterns; GLPG: *Ganoderma lucidum* polysaccharide; NLRP3: NOD-like receptor protein 3; PAMPs: pathogen-associated molecular patterns. Created in BioRender. Bijak, M. (2025) https://BioRender.com/vn0tamv (accessed on 27 October 2025).

In a study conducted by Chen et al., GLPG was investigated for its effects on NLRP3 inflammasome activation in a CCL_4_-induced liver injury model. The authors measured protein levels of NLRP3, ASC, and caspase-1, key components of the inflammasome complex responsible for IL-1β and IL-18 maturation. GLPG supplementation significantly reduced the expression of these proteins compared to untreated mice, suggesting a suppression of inflammasome activation [109]. The inflammasome is closely linked to pyroptosis, a newly recognized form of programmed cell death, whose activation pathway may be dependent on caspase-1 [131]. By inhibiting NLRP3, GLPG may potentially reduce hepatocyte pyroptosis and limit the release of pro-inflammatory cytokines, thereby attenuating liver inflammation and tissue damage. Although this mechanism remains hypothetical, it highlights the potential interplay between multiple molecular pathways and cellular processes in hepatoprotection. The effect of fungal polysaccharide, exemplified by GLPG, on the NLRP3 inflammasome is presented in Figure 3.

Downregulation of NLRP3 was also shown for the lentinan in the study by Yang et al. on arsenic-induced hepatotoxicity in mice [112] and in Zhang et al. study, in which *Nlrp3* on CCL_4_-induced liver injury was significantly diminished after AAP supplementation [29].

The NLRP3 inflammasome is a key molecular complex that links metabolic stress, oxidative damage, and chronic inflammation in liver disease progression. Fungal polysaccharides exhibit potent anti-inflammatory effects by inhibiting inflammasome activation through both direct and indirect mechanisms. They suppress the initial priming signal mediated by NF-kB and block the secondary activation signal. This inhibition prevents inflammasome assembly, the cleavage of procaspase-1 to active caspase-1, and the maturation of pro-inflammatory cytokines, including IL-1β and IL-18. Nevertheless, the current state of knowledge reveals gaps in the quantity and depth of research required to unequivocally confirm the significant role of fungal polysaccharides in modulating the NLRP3 inflammasome. This area necessitates intensive, targeted investigation to fully harness its therapeutic potential.

## 7. Challenges and Future Perspectives

### 7.1. Limitations

While the body of evidence supporting the hepatoprotective, antioxidant, and immunomodulatory effects of fungal polysaccharides continues to expand, their clinical translation—either as nutraceuticals or therapeutics—faces several scientific, technical, and regulatory constraints. Native fungal polysaccharides are often high-molecular-weight, hydrophilic, and susceptible to partial degradation by gut enzymes and the microbiota. Consequently, their systemic absorption and hepatic exposure after oral dosing are typically limited and variable. Polysaccharides represent biomolecules of considerably higher structural complexity compared with, e.g., nucleic acids or proteins, owing to their ability to form glycosidic bonds at multiple positions of each monosaccharide unit. This flexibility enables the generation of both linear and highly branched architectures. Oral administration of such structurally complex compounds requires overcoming numerous barriers—biological, chemical, immunological, and mechanical. The challenge is further compounded by analytical difficulties, since the physicochemical nature of polysaccharides, including their lack of ultraviolet absorption and fluorescent groups, limits the use of conventional detection techniques [132]. Studies analyzed in this review exhibit substantial variability in experimental design, including differences in animal species, liver injury model, doses, extraction methods, and duration of treatment. Those variables further complicate translational prioritization. Most of the evidence is derived from preclinical in vitro and animal studies, with a lack of robust clinical data confirming efficacy and safety in humans. Only a few clinical or translational investigations, such as those involving pleuran, PSK, and lentinan, have been reported, and they focus mainly on immune function rather than liver-specific outcomes.

Moreover, their biological activity is strongly dependent on molecular weight, degree of branching, conformation, presence of impurities, pharmacokinetics, and other physical and chemical characteristics of a particular polysaccharide. Unfortunately, not all reports provide comprehensive structural and functional characterization. The composition and molecular characteristics of polysaccharide preparations often differ between studies, which hinders reproducibility and direct comparison of results. Researchers often evaluate heterogeneous fractions of polysaccharides isolated from a single mushroom species or even from specific anatomical parts like fruiting bodies or mycelium, rather than investigating a well-defined compound such as lentinan. This practice hampers reproducibility and makes interstudy comparisons challenging. Investigations of molecular pathways, such as TLR4/NF-κB or Nrf2, are frequently performed in a fragmentary manner and do not provide a full, comprehensive mechanistic understanding. Thus, there is an urgent need for standardized in vivo models with harmonized endpoints to enable more robust interpretation of findings and accelerate the path toward clinical translation. 

### 7.2. Prospects

To overcome these limitations, several advanced delivery strategies merit exploration. Among them, nano- or micro- particulate delivery systems (e.g., polysaccharide-based nanoparticles, polymeric micelles, liposomes, solid lipid nanoparticles) offer the potential to enhance absorption, protect from enzymatic degradation, and target delivery to the liver or tumor microenvironment. In the study conducted by Chen et al., Mulberry leaf polysaccharide (MLP) was covalently conjugated to the liposomal surface, creating an MLP-caged liposome system, which improved its structural integrity in the gastrointestinal tract and increased intestinal accumulation, thus increasing bioavailability by omitting hepatic first-pass metabolism and absorbing through lymphatic pathways. Moreover, this conjugation expanded the sustained release time and improved transport across cells in comparison to conventional encapsulated liposomes [133]. In the study conducted by Sun et al., *Angelica sinensis* polysaccharide was conjugated with deoxycholic acid to form a nanoparticle able to carry oridonin, enhancing its potential for application as a new drug carrier [134]. The concept of polysaccharides as polysaccharide-based drug delivery systems has been comprehensively reviewed by Hsu et al. [135]. Furthermore, Liu et al. demonstrated that lentinan-functionalized selenium nanoparticles (LNT-SeNPs) can serve as a novel label-free theranostic platform, combining both therapeutic and imaging capacities. In a performed study, Lentinan not only provided functional surface coating but also enabled Chemical Exchange Saturation Transfer in Magnetic resonance imaging (CEST MRI) detection through its hydroxyl protons, allowing for image-guided delivery without additional chemical labeling, thus highlighting the potential of LNT-SeNPs as a biocompatible and translationally feasible nanoplatform able to overcome delivery barriers [136].

Fungal polysaccharides hold considerable promise as versatile tools in nanomedicine, functioning not only as bioactive agents but also as functional carrier materials. Their inherent biocompatibility, biodegradability, and immunomodulatory capacity support a wide range of applications, ranging from cancer therapeutics and immune modulation to the regulation of inflammatory states and applications in metabolic support. Nevertheless, their therapeutic potential is often constrained by poor solubility and limited bioavailability. Advances in nanotechnology, including encapsulation within nanoparticles, nanogels, and nanoemulsions, provide effective strategies to overcome barriers. When employed as carrier matrices, fungal polysaccharides can facilitate efficient incorporation of therapeutic agents, allow surface modifications that improve targeting, and interact with the immune system in ways that enhance therapeutic outcomes. These combined features support controlled release, increased stability, and synergistic action in co-therapies, underscoring the translational potential of fungal polysaccharide-based nanoplatforms in future biomedical research and clinical practice [137].

## 8. Conclusions

Fungal polysaccharides constitute a promising class of multifunctional biopolymers that exert hepatoprotective effects through the integrated modulation of inflammatory, oxidative, and metabolic pathways. Evidence from in vitro and in vivo studies consistently demonstrates their capacity to restore homeostasis, suppress NF-kB-driven inflammatory signaling, activate the Nrf2 antioxidant defense system, and attenuate NLRP3 inflammasome activation. Moreover, their influence on the gut-liver axis, manifested through microbiota modulation and reinforcement of intestinal barrier integrity, provides an additional systemic mechanism contributing to liver protection.

Despite the growing mechanistic understanding, the translation of fungal polysaccharides from experimental studies to clinical applications remains limited. To realize their full therapeutic potential, future research should prioritize standardized characterization of polysaccharide fractions. Provide reproducible animal models and well-designed clinical trials addressing bioavailability, safety, and pharmacokinetics. Integration of nanotechnological delivery systems may further enhance their efficacy by improving absorption, stability, and targeting.

Collectively, fungal polysaccharides represent a promising frontier in hepatoprotection—bridging nutritional, pharmacological, and technological approaches. Their multifunctional nature supports the concept of translational nutraceuticals, capable of both preventing and mitigating liver injury through coordinated molecular and systemic regulation. Continued interdisciplinary research is essential to transform these natural macromolecules from experimental bioactives into clinically relevant therapeutics for liver health.

## Figures and Tables

**Table 1:** The overview of selected fungal polysaccharides.

Polysaccharide	Family	Fungi	Structure/Composition	Molecular Weight	References
*Auricularia auricula* polysaccharide (AAP)	heteropolysaccharide	*Auricularia auricula*	monosaccharides: glucose, arabinose, fucose, mannose, rhamnose, galactose, xylose	1.63 × 10^6^ Da	[29]
*Antrodia cinnamomea* polysaccharide (ACP)	heteropolysaccharide	*Antrodia cinnamomea*	D-glucan (76.3%)	-	[30]
Chitin	homoglycan	*Mucor rouxii* *Aspergillus niger* *Lentinus edodes*	N-acetyl-D-glucosamine and D-glucosamine units	50–300 kDa	[31]
*Coriolus versicolor* polysaccharide (CVP)	heteropolysaccharide	*Coriolus versicolor*	monosaccharides: mannose, glucose, galactose, xylose, fucose; glucuronic acid (→1)-β-D-Man-(6,4→1)-α-D-Gal-(3→1)-α-D-Man-(4→1)-α-D-Gal-(6→) backbone [(→1)-α-D-Glc-(6→1)-α-D-Man-(4,3→1)-β-D-Xyl-(2→1)-β-D-Glc] (O-6 position) and [(→1)-α-D-Fuc-(4→1)-α-D-Man] (O-4 position) branches	17,478 Da	[32]
*Dictyophora indusiata* polysaccharide (DIP)	heteropolysaccharide	*Dictyophora indusiata*	monosaccharides: glucose, galactose, mannose, xylose; (→3)-Glcp-(1→, →4)-Glcp-(1→, →3,4)-Glcp-(→1→, →3,4)-Galp-(1→); branches at (→6)-Manp-(1→)	1132 Da	[33,34]
*Cordyceps militaris* polysaccharides (EPCM)	heteropolysaccharide	*Cordyceps militaris*	monosaccharides: mannose, ribose, rhamnose, glucuronic acid, galacturonic acid, N-acetyl-glucosamine, N-acetyl-galactosamine, glucose, galactose, xylose, arabinose, fucose (→3)-α-L-Fucp(1→, →4)-α-D-Glcp-(1→, →2,6)-α-D-Galp-(1→, →3)-α-Glcp-(1→, →6)-β-D-Galp-(1→) and β-D-Manp-(1→)	20,792 Da	[35]
*Fomitopsis pinicola* mycelial polysaccharides (FPMPS)	heteropolysaccharide	*Fomitopsis pinicola*	myo-inositol, fucose, galactose, glucose, mannose, fructose	-	[36,37]
*Grifola frondosa* polysaccharides (GFPs)	heteropolysaccharide	*Grifola frondosa*	varies with fraction monosaccharides: predominantly mannose, glucosamine, glucose, galactose, fucose GFP-N1:1 →3, 1→4, and 1→6 glycosidic bonds GFP-N2:1→2, 1→3, 1→4, and 1→6 glycosidic bonds	varies with fraction GFP-N1: 3.323 × 10^3 ^ kDa GFP-N2: 10.8 kDa	[38,39,40]
*Ganoderma lucidum* proteoglycan (GLPG)	proteoglycan	*Ganoderma lucidum*	carbohydrate: protein ratio of 10.4:1	-	[41,42]
*Hirsutella sinensis* polysaccharides (HSP)	heteropolysaccharide	*Hirsutella sinensis*	varies with fraction HSWP-1a: α-(1,4)-D-glucan HSWP-1b: mainly mannoglucans with a 1,4-Glc/1,4,6-Man backbone and 1-linked Glc side chains at O-6 of 1,4-Glc HSWP-1c: mainly galactomannoglucans HSWP-1d: mainly mannoglucans with a 1,4-Glc/1,4,6-Man backbone and 1-linked Glc side chains at O-6 of 1,4-Glc HSP-III: mannose, galactose, rhamnose, arabinose, xylose, glucose; majorly composed of (1→3) glucose	varies with fraction fraction H1: >300 kDa fraction HSP-III: 513.90 kDa	[43,44,45]
Intracellular mycelium polysaccharides from *Pleurotus geesteranus* (IMPP)	heteropolysaccharide	*Pleurotus geesteranus*	monosaccharides: fucose, arabinose, xylose, mannose, galactose, glucose	-	[46]
Lentinan	β-glucan	*Lentinula edodes*	β(1→3) backbone of D-glucose units with two β(1→6) D-glucosyl residues	146–504 kDa	[47]
*Morchella exculenta* polysaccharide (MCP)	heteropolysaccharide	*Morchella exculenta*	glucose, mannose, galactose	1.69 × 10^5^ Da	[48]
*Morchella esculenta* polysaccharide 2 (MEP2)	heteropolysaccharide	*Morchella esculenta*	monosaccharides: glucose, galactose, mannose, glucuronic acid (→4)-α-D-Glcp-(1→) glucan backbone with α-D-Glcp-(1→4)-α-D-Glcp-(1→) residue and an α-D-Glcp-(1→) residue at H-6 position	959 kDa	[49]
*Poria cocos* polysaccharides (PCP)	β-glucan	*Poria cocos*	monosaccharides: glucose, fucose, arabinose, xylose, mannose, galactose β-(1→3)-linked glucose backbone with β-(1→6)-linked glucose side chains	4.1 × 10^4^ to 5 × 10^6^ Da	[50]
Polysaccharide isolated from *Pleuroteus geestranus* (PFP-1)	heteropolysaccharide	*Pleuroteus geestranus*	monosaccharides: fucose, arabinose, galactose, glucose, xylose, mannose, ribose pyranose-polysaccharide in a triple-helical conformation linked by t-β-Glcp, 1,6-α-Glcp and 1,2,6-α-Galp	15.5 kDa	[51]
*Phellinus linteus* polysaccharides (Phps)	heteropolysaccharide	*Phellinus linteus*	monosaccharides: glucose, mannose, galactose, N-acetylglucosamine β-(1→3) glycosidic bonds in backbone with (1→6) branches	22–1700 kDa	[52,53]
Pleuran	β-glucan	*Pleurotus ostreatus*	β-(1→3)-linked D-glucopyranosyl units branched at the O-6 position every fourth glucose residue	600–700 kDa	[54,55]
Polysaccharide-K (PSK)	Protein-bound polysaccharide	*Trametes versicolor*	mainly glucose with minor amounts of mannose, fucose, xylose, and galactose peptide fraction: glutamic acid, aspartic acid, leucine, valine, threonine, serine, glycine β-glucan backbone with (1→4) linkages, branched at the 3- and 6-positions, and incorporates (1→3) and (1→6) glucopyranosidic bonds	94 kDa	[56]
Polysaccharopeptide (PSP)	Protein-bound polysaccharide	*Trametes versicolor* (syn. *Coriolus versicolor*)	glucose with predominant α-1,4 and β-1,3 glucosidic linkages; arabinose and rhamnose peptide fraction: rich in aspartic acid and glutamic acid	100 kDa	[57]
Polysaccharides extracted from the water extract of *Ganoderma lucidum* (WEGL)	heteropolysaccharide	*Ganoderma lucidum*	G1: mannose, glucose, galactose, glucoctosamine, arabinose, galactosamine, rhamnose, fucose	varies with fraction G1: high molecular weight polysaccharides (>300 kDa) G2: 190,399 kDa G3: <10 kDa	[58]

## Abbreviations

The following abbreviations are used in this manuscript:

AAP	*Auricularia auricula* polysaccharide
Acc	Acetyl-CoA Carboxylase
ACP	*Antrodia cinnamomea* polysaccharide
ADH	alcohol dehydrogenase
AKT	Protein kinase B
ALD	alcohol-related liver disease
ALDH	acetal dehydrogenase
ALP	alkaline phosphatase
ALT	alanine aminotransferase
AMPK	AMP-activated protein kinase
APAP	Acetaminophen
AREs	antioxidant response elements
ASC	apoptosis-associated speck-like protein containing a caspase recruitment domain CARD
AST	aspartate aminotransferase
BAX	Bcl-2-associated X protein
Bcl-2	B-cell lymphoma 2
Cat	catalase
CCL_4_	C-C Motif Chemokine Ligand 4
Cd14	Cluster of Differentiation 14
ChNPs	chitosan-based nanoparticles
Cox2	Cyclooxygenase-2
CVP	*Coriolus versicolor* polysaccharide
Cyp	cytochrome P450
DAMPs	damage-associated molecular patterns
DIP	*Dictyophora indusiata* polysaccharide
DPPH	2,2-diphenyl-1-picrylhydrazyl
EnPs	endopolysaccharides
EPCM	Enzymatic-extractable polysaccharides from *Cordyceps militaris*
FPMPS	*Fomitopsis pinicola* mycelial polysaccharides
GCLC	Glutamate–Cysteine Ligase Catalytic Subunit
GCLM	Glutamate–Cysteine Ligase Modifier Subunit
GFP	*Grifola frondosa* polysaccharide
GGT	Gamma-Glutamyl Transferase
GLPG	*Ganoderma lucidum* proteoglycan
GPx4	glutathione peroxidase 4
GSH-Px	glutathione peroxidase
GSK3β	cascade glycogen synthase kinase 3 beta
HCC	hepatocellular carcinoma
HFD	high-fat diet
HO-1	Heme Oxygenase-1
HSCs	hepatic stellate cells
HSP	*Hirsutella sinensis* polysaccharide
IFNα	interferon α
IL	interleukin
IMPP	intracellular mycelium polysaccharides from *Pleurotus geesteranus*
iNos	Inducible Nitric Oxide Synthase
Ifnγ	Interferon gamma
IPS	intracellular polysaccharide
JNK	c-Jun N-terminal kinase
KCs	Kupffer cells
Lbp	Lipopolysaccharide Binding Protein
LDH	lactate dehydrogenase
LDL-C	low-density lipoprotein cholesterol
LPO	lipid peroxidation
LPS	lipopolysaccharide
LSECs	liver sinusoidal endothelial cells
MAFLD	metabolic dysfunction-associated fatty liver disease
MCP	*Morchella esculenta* polysaccharide
MCP-1	Monocyte Chemoattractant Protein-1
MDA	malondialdehyde
MEP2	*Morchella esculenta* polysaccharide 2
MPO	myeloperoxidase
MyD88	Myeloid differentiation primary response 88
NASH	Non-Alcoholic Steatohepatitis
NF-κB	Nuclear Factor kappa-light-chain-enhancer of activated B cells
NLRP3	NOD-like receptor protein 3
NQO1	NAD(P)H quinone oxidoreductase 1
Nrf2	Nuclear factor erythroid 2–related factor 2
PAMPs	pathogen-associated molecular patterns
PCP	*Poria cocos* polysaccharides
Phps	*Phellinus linteus* polysaccharides
PINK1	PTEN-indiced kinase 1
PI3K	Phosphoinositide 3-Kinase
p-NF-κB p65	phosphorylated NF-κB, p65 subunit
p-IκBα	phosphorylated inhibitor of kappa B alpha
Pparα	Peroxisome Proliferator-Activated Receptor Alpha
PRRs	pattern recognition receptors
PSK	polysaccharide-K
PSP	polysaccharopeptide
Ptp1b	Protein Tyrosine Phosphatase 1B
PUFAs	polyunsaturated fatty acids
RGD	Arginylglycylaspartic acid
ROS	reactive oxygen species
RR	response rates
Sirt1	sirtuin 1
Socs2	Suppressor of Cytokine Signaling 2
SOD	superoxide dismutase
T-AOC	Total Antioxidant Capacity
TLR4	Toll-like receptor 4
TNFα	tumor necrosis factor α
WEGL	polysaccharides from the water extract of *Ganoderma lucidum*
